# Protein Encapsulation: A Nanocarrier Approach to the Fluorescence Imaging of an Enzyme-Based Biomarker

**DOI:** 10.3389/fchem.2020.00389

**Published:** 2020-06-03

**Authors:** Zhiyuan Jia, Hai-Hao Han, Adam C. Sedgwick, George T. Williams, Lauren Gwynne, James T. Brewster, Steven D. Bull, A. Toby A. Jenkins, Xiao-Peng He, Holger Schönherr, Jonathan L. Sessler, Tony D. James

**Affiliations:** ^1^Department of Chemistry and Biology, Physical Chemistry & Research Center of Micro- and Nanochemistry and Engineering (Cμ), University of Siegen, Siegen, Germany; ^2^Key Laboratory for Advanced Materials and Joint International Research Laboratory of Precision Chemistry and Molecular Engineering, Feringa Nobel Prize Scientist Joint Research Center, School of Chemistry and Molecular Engineering, East China University of Science and Technology, Shanghai, China; ^3^Department of Chemistry, The University of Texas at Austin, Austin, TX, United States; ^4^Department of Chemistry, University of Bath, Bath, United Kingdom

**Keywords:** elastase detection, BSA-based nanocarrier, nanocarrier-based enzyme detection, fluorescence imaging, cell imaging

## Abstract

Here, we report a new pentafluoropropanamido rhodamine fluorescent probe (ACS-HNE) that allows for the selective detection of neutrophil elastase (NE). ACS-HNE displayed high sensitivity, with a low limit of detection (<5.3 nM), and excellent selectivity toward elastase over other relevant biological analytes and enzymes. The comparatively poor solubility and cell permeability of neat ACS-HNE was improved by creating an ACS-HNE-albumin complex; this approach allowed for improvements in the *in situ* visualization of elastase activity in RAW 264.7 cells relative to ACS-HNE alone. The present study thus serves to demonstrate a simple universal strategy that may be used to overcome cell impermeability and solubility limitations, and to prepare probes suitable for the cellular imaging of enzymatic activity *in vitro*.

## Introduction

Fluorescent probes have found widespread application in imaging biomarkers relevant to physio- and pathological cellular pathways (Kobayashi et al., 2010; Chan et al., 2012; Wu et al., 2017; Erbas-Cakmak et al., 2018; Sedgwick et al., 2018d). Within this paradigm, reaction-based systems containing an integrated reactive motif offer specificity in probing biochemical processes with concomitant utility as a diagnostic tool for medical applications (Caramello et al., 1993; Burgos-Barragan et al., 2017; Xiao et al., 2018; Akashi et al., 2019). Common dye scaffolds are, however, still limited and generally rely upon flat polyaromatic frameworks. Such systems are typically restricted by their poor solubility and cell permeability. In an attempt to overcome these limitations, supramolecular nanocarrier constructs have been devised in an effort to enhance solubility, photophysical properties, and chemoselectivity (Dondon and Fery-Forgues, 2001; Sheng et al., 2014; Chen and Liu, 2016; Fu et al., 2018; Yan et al., 2018; Gao et al., 2019; Miranda-Apodaca et al., 2019).

Continued advances in imaging methods coupled with fluorescent probe technologies have made real-time monitoring of enzymatic activity a viable tool for understanding fundamental biological processes (Liu et al., 2018; Yang et al., 2019). Our own efforts have focused on the development of fluorescent-based probes for the detection of biologically relevant species that are thought to be intimately involved in a number of pathological processes, such as inflammation, neurodegenerative diseases and cancer (Sedgwick et al., 2017, 2018a,b,c,d; Wu et al., 2017, 2018; Odyniec et al., 2018; Gwynne et al., 2019). In the context of these efforts, we turned our attention toward the detection of the enzyme neutrophil elastase (NE). NE is a serine protease primarily secreted by neutrophils during an inflammatory response. NE possesses important protective functions, which include the remodeling of the extracellular matrix. It also acts as a host defense against bacterial infections. NE is found in inflamed tissues and wound exudate (Mitra et al., 2013). Elevated levels of NE have been associated with a number of inflammatory-related diseases, such as chronic obstructive pulmonary disease (COPD), cystic fibrosis (CF), acute lung injury (ALI), and acute respiratory distress syndrome (ARDS) (Belaaouaj et al., 1998; Shapiro et al., 2003; Sly et al., 2009; Korkmaz et al., 2010). In order to utilize NE as a potential therapeutic target for disease treatments, new systems elucidating its function in disease are essential (Henriksen and Sallenave, 2008; Ho et al., 2014).

Current methods for determining NE activity utilize a combination of indirect separation methods (i.e. HPLC or LC–MS) and direct electrochemical, UV-Vis spectroscopic, or fluorescence-based probes (Bieth et al., 1974; Wang et al., 2008; González-Fernández et al., 2018). These latter optical methods have proven useful for monitoring NE, but remain cost prohibitive due to the use of peptide-based substrates (Wang et al., 2008; Gehrig et al., 2012; Kasperkiewicz et al., 2014; Schulz-Fincke et al., 2018). Yang and co-workers recently reported a simple non-peptide-based strategy for the selective detection of elastase (Sun et al., 2013). Their coumarin-derived fluorescent probe was functionalised with a reactive pentafluoropropionamide unit, which served as a substrate for NE that, in turn, served to unveil the activated fluorescent dye. Unfortunately, the short excitation wavelength characteristic of many coumarin-systems limited the utility of this system as tool for monitoring elastase activity, and apparently precluded cellular imaging experiments.

**Scheme 1:**
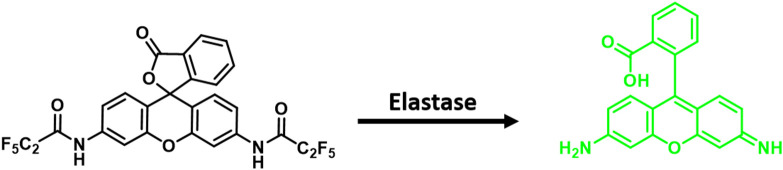
Development of a rhodamine fluorescent probe for the detection of neutrophil elastase (NE).

Here we report a non-peptide rhodamine fluorescent probe for the detection of elastase (**ACS-HNE**)—Scheme 1. This new system is based on the use of a rhodamine-based fluorophore. This was considered attractive from a design perspective since this fluorophore core is readily subject to synthetic modification. Moreover, rhodamine derivatives typically display high fluorescent quantum yields and display photophysical properties appropriate for *in vitro* study (Chan et al., 2012; Bhuniya et al., 2014). However, as true for many near-planar dye systems, probes based on rhodamine often suffer from poor solubility or a tendency to aggregate in aqueous milieus. In the present instance, we have built upon recent protein-based nanocarrier strategies (Han et al., 2020), to create an **ACS-HNE/ Bovine Serum Albumin (BSA**) hybrid that displays enhanced solubility relative to **ACS-HNE** and which acts as a fluorescent probe that allows for enzyme-based imaging in RAW 264.7 cells. Despite the recent report of an NIR probe for NE detection *in vitro* and *in vivo* (Liu et al., 2019), we believe the rhodamine scaffold of **ACS-HNE** offers an excellent platform for further derivatisation. In addition, this BSA-nanocarrier system represents a global strategy for researchers to overcome the solubility issues associated with hydrophobic fluorescent imaging agents designed to detect enzyme-based biomarkers.

## Results and Discussion

### Chemistry

Briefly, **ACS-HNE** was synthesized in one step from commercially available rhodamine 110 (RH110) by dissolving in *N,N*-dimethylformamide, deprotonating with sodium hydride at 0°C, and acylating with pentafluoropropionic anhydride. The product was isolated, after purification by column chromatography, in 43% yield—Scheme S1.

### Spectroscopic Studies of ACS-HNE

**Figure 1:**
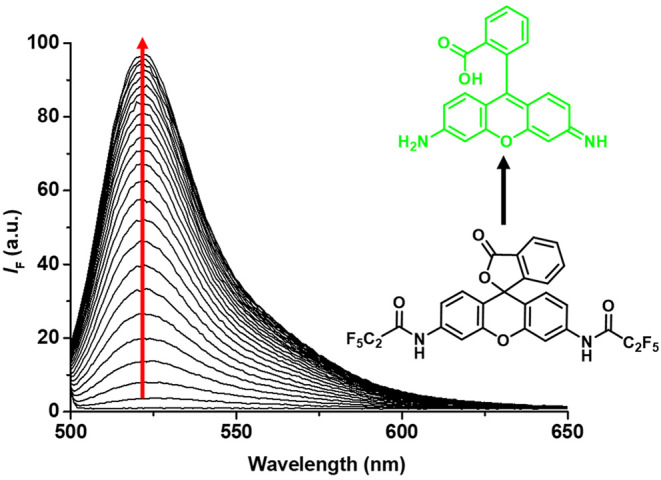
Fluorescence emission spectra of **ACS-HNE** (5 μM) over time (24 h) in buffered elastase enzyme (2 μM) solution (in PBS buffer, pH = 7.40); λ_ex_ = 496 nm.

With **ACS-HNE** in hand, UV-Vis and fluorescence spectroscopic experiments were carried out to evaluate whether this putative probe could be used to monitor NE activity. As shown in Figures S1, S2, the addition of elastase (2 μM in PBS) led to a large increase in UV-Vis absorption at ~490 nm, as would be expected for the enzyme-based release of rhodamine 110 in accord with Scheme 1. A strong increase in the fluorescence intensity (*I*_F_) was also observed after the addition of elastase (2 μM in PBS)—Figure 1. Dose-response studies involving fluorescence monitoring revealed a linear increase in emission intensity with increasing enzyme concentration (Figures S3, S4). Such behavior is fully consistent with the expected Michaelis–Menten kinetics (Nelson and Cox, 2005). Limit of detection (LoD) values for NE using **ACS-HNE** were calculated using the assay developed by Schönherr and co-workers (Sadat Ebrahimi et al., 2015).

The apparent pseudo-first order rate constant for the reaction (Figure S4) and the LoD value for the detection of RH110 (Figure S5) were determined to be 0.3 min^−1^μM^−1^ and 1.7 nM, respectively. At set times, **ACS-HNE** demonstrated high sensitivity toward elastase. For instance, LoD values of 5.3 and 2.6 nM were calculated at 60 and 120 min, respectively (Figure S6). These low LoD values were comparable to previously reported elastase detection systems (Sun et al., 2013; Ebrahimi et al., 2015; Liu et al., 2019). The selectivity of **ACS-HNE** was also tested by treating it with other potentially competing enzymes and biologically relevant analytes. As illustrated in Figure S7, **ACS-HNE** displayed excellent selectivity for elastase over a number of potential interferants, including trypsin, glutathione (GSH), and hydrogen peroxide (H_2_O_2_).

### Cellular Imaging of ACS-HNE and ACS-HNE/BSA

**Figure 2:**
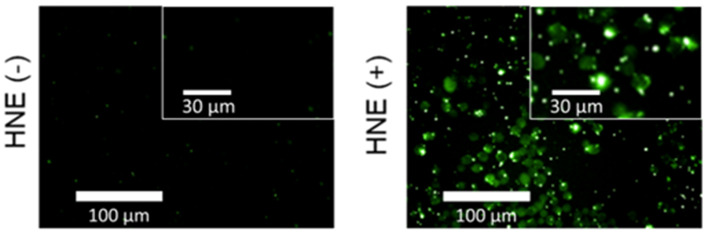
Widefield fluorescence micrographs of RAW 264.7 cells incubated with **ACS-HNE** (20 μM) before (–) or after (+) addition of human NE (HNE, 4 ng μL^−1^, 100 μL); Excitation and emission wavelengths for **ACS-HNE** are 460–490 nm and 500–550 nm, respectively. The insert shows magnified sections of the corresponding fluorescence micrograph.

In light of the excellent selectivity for NE displayed by **ACS-HNE**, we turned our attention toward evaluating it as a potential probe for cellular imaging. Predicative cytotoxicity experiments involving **ACS-HNE** revealed minimal cytotoxicity, which we took as a favorable augury for cellular imaging experiments (Figure S8). As shown in Figure 2, no initial fluorescence emission was observed in RAW 264.7 cells when incubated with **ACS-HNE** (20 μM). Upon exposure to exogenous elastase (4 ng μL^−1^, 100 μL), a substantial increase in the fluorescence intensity was observed. Not surprisingly, due to the high lipophilicity of the dye scaffold, **ACS-HNE** displayed poor cell permeability with resultant precipitation, as seen by the fluorescent “spots” around each cell.

**Figure 3:**
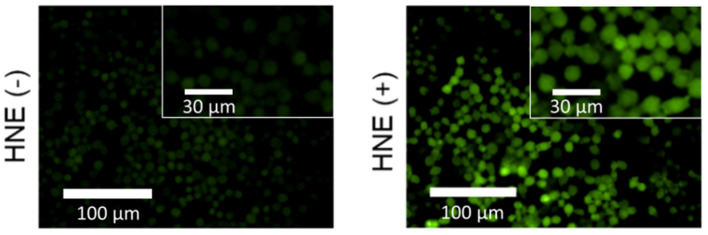
Widefield fluorescence micrographs of RAW 264.7 cells incubated with **ACS-HNE/BSA** (20/100 μM) before (–) or after (+) addition of human NE (HNE, 4 ng μL^−1^, 100 μL); Excitation and emission wavelengths for **ACS-HNE** are 460–490 nm and 500–550 nm, respectively. The inset shows magnified sections of the corresponding fluorescence micrograph.

To overcome the issues of poor cell permeability and solubility, we applied a protein nanocarrier encapsulation strategy that involved treatment with the natural transport protein BSA. Previous studies have demonstrated BSA as an attractive candidate for the targeted intracellular delivery of therapeutics (Karami et al., 2020). Therefore, we rationalized that the use of BSA would overcome these current limitations by facilitating the effective cellular uptake of **ACS-HNE**. In accordance with the previously reported protocol, **ACS-HNE** was mixed with BSA at a molar ratio of 1:5 (**ACS-HNE/BSA** = 20 μM/100 μM) prior to carrying out cellular imaging. The resulting **ACS-HNE/BSA** hybrid was subsequently added to the cells. As can be seen in Figure 3 and Figure S9, a low background fluorescence intensity was observed. The subsequent addition of exogenous elastase (154.4 nM, 100 μL) resulted in a 2.5-fold increase in the fluorescence intensity with little evidence of precipitation. This increase in fluorescence was attributed to the cellular uptake of exogenous elastase (Houghton et al., 2010) and reaction with **ACS-HNE**. This level of enhancement demonstrates the effective enzyme-based imaging of probe-albumin complexes and highlights the utility of this method as a means to increase solubility and cellular uptake for probes whose utility might otherwise be limited.

## Conclusions

In summary, we have developed a novel rhodamine-based fluorescent probe (**ACS-HNE**) prepared using a straightforward one-step reaction procedure. Synthetic attachment of the pendant pentafluoropropionamide functionality to a rhodamine core endows **ACS-HNE** with high sensitivity and selectivity toward elastase. This, in turn, permitted quantification with a low limit of detection (5.3 nM at an observation time of 60 min). The comparatively low cell permeability and poor solubility of native **ACS-HNE** was enhanced using a protein nanocarrier-based strategy involving formation an **ACS-HNE/BSA** hybrid prior to cell imaging. The combination of fluorescent probe development and nanocarrier solubilization has facilitated the development of new class of non-peptide-based fluorescent probes suitable for monitoring elastase activity *in vitro* (Bhuniya et al., 2014). We are currently extending the reaction-based fluorescence modulation and nanoparticle solubilisation approach to create new *in vitro* enzyme-specific sensor systems.

## Data Availability Statement

All datasets generated for this study are included in the article/Supplementary Material.

## Author Contributions

ZJ and AS carried out synthetic and spectroscopic experiments and co-wrote the manuscript with H-HH who also carried out the cellular imaging experiments. GW, LG, and JB carried out background experiments and helped with the preparation of the manuscript. X-PH and AJ are supervisors of H-HH, GW, and LG. SB, HS, JS, and TJ all conceived the idea and helped with the manuscript.

## Conflict of Interest

The authors declare that the research was conducted in the absence of any commercial or financial relationships that could be construed as a potential conflict of interest.

## References

[B1] Akashi T. Isomoto H. Matsushima K. Kamiya M. Kanda T. Nakano M.. (2019). A novel method for rapid detection of a Helicobacter pylori infection using a γ-glutamyltranspeptidase-activatable fluorescent probe. Sci. Rep. 9:9467. 10.1038/s41598-019-45768-x 31263136 PMC6603024

[B2] Belaaouaj A. McCarthy R. Baumann M. Gao Z. Ley T. J. Abraham S. N.. (1998). Mice lacking neutrophil elastase reveal impaired host defense against gram negative bacterial sepsis. Nat. Med. 4, 615–618. 10.1038/nm0598-615 9585238

[B3] Bhuniya S. Maiti S. Kim E. Lee H. Sessler J. L. Hong K. S.. (2014). An activatable theranostic for targeted cancer therapy and imaging. Angew. Chemie Int. Ed. 53, 4469–4474. 10.1002/anie.201311133 24644015

[B4] Bieth J. Spiess B. Wermuth C. G. (1974). The synthesis and analytical use of a highly sensitive and convenient substrate of elastase. Biochem. Med. 11, 350–357. 10.1016/0006-2944(74)90134-3 4429553

[B5] Burgos-Barragan G. Wit N. Meiser J. Dingler F. A. Pietzke M. Mulderrig L.. (2017). Mammals divert endogenous genotoxic formaldehyde into one-carbon metabolism. Nature 548, 549–554. 10.1038/nature23481 28813411 PMC5714256

[B6] Caramello P. Lucchini A. Savoia D. Gioannini P. (1993). Rapid diagnosis of malaria by use of fluorescent probes. Diagn. Microbiol. Infect. Dis. 17, 293–297. 10.1016/0732-8893(93)90038-9 8112044

[B7] Chan J. Dodani S. C. Chang C. J. (2012). Reaction-based small-molecule fluorescent probes for chemoselective bioimaging. Nat. Chem. 4, 973–984. 10.1038/nchem.1500 23174976 PMC4096518

[B8] Chen Q. Liu Z. (2016). Albumin carriers for cancer theranostics: a conventional platform with new promise. Adv. Mater. 28, 10557–10566. 10.1002/adma.201600038 27111654

[B9] Dondon R. Fery-Forgues S. (2001). Inclusion complex of fluorescent 4-hydroxycoumarin derivatives with native β-cyclodextrin: enhanced stabilization induced by the appended substituent. J. Phys. Chem. B 105, 10715–10722. 10.1021/jp010900h

[B10] Ebrahimi M. M. S. Laabei M. Jenkins A. T. A. Schönherr H. (2015). Autonomously sensing hydrogels for the rapid and selective detection of pathogenic bacteria. Macromol. Rapid Commun. 36, 2123–2128. 10.1002/marc.201500485 26474087

[B11] Erbas-Cakmak S. Kolemen S. Sedgwick A. C. Gunnlaugsson T. James T. D. Yoon J.. (2018). Molecular logic gates: the past, present and future. Chem. Soc. Rev. 47, 2228–2248. 10.1039/C7CS00491E 29493684

[B12] Fu Y. Han H.-H. Zhang J. He X.-P. Feringa B. L. Tian H. (2018). Photocontrolled fluorescence “double-check” bioimaging enabled by a glycoprobe–protein hybrid. J. Am. Chem. Soc. 140, 8671–8674. 10.1021/jacs.8b05425 29940117

[B13] Gao S. Wei G. Zhang S. Zheng B. Xu J. Chen G.. (2019). Albumin tailoring fluorescence and photothermal conversion effect of near-infrared-II fluorophore with aggregation-induced emission characteristics. Nat. Commun. 10, 1–15. 10.1038/s41467-019-10056-9 31101816 PMC6525245

[B14] Gehrig S. Mall M. A. Schultz C. (2012). Spatially resolved monitoring of neutrophil elastase activity with ratiometric fluorescent reporters. Angew. Chemie Int. Ed. 51, 6258–6261. 10.1002/anie.201109226 22555935

[B15] González-Fernández E. Staderini M. Yussof A. Scholefield E. Murray A. F. Mount A. R.. (2018). Electrochemical sensing of human neutrophil elastase and polymorphonuclear neutrophil activity. Biosens. Bioelectron. 119, 209–214. 10.1016/j.bios.2018.08.013 30138864

[B16] Gwynne L. Sedgwick A. C. Gardiner J. E. Williams G. T. Kim G. Maillard J. Y.. (2019). Long wavelength TCF-based fluorescence probe for the detection of Alkaline Phosphatase in live cells. Front. Chem. 7:255. 10.3389/fchem.2019.00255 31119120 PMC6508040

[B17] Han H. H. Sedgwick A. C. Shang Y. Li N. Liu T. Li B. H. (2020). Protein encapsulation: a new approach for improving the capability of small-molecule fluorogenic probes. Chem. Sci. 11, 1107–1113 10.1039/C9SC03961A PMC8145178 34084367

[B18] Henriksen P. A. Sallenave J.-M. (2008). Human neutrophil elastase: mediator and therapeutic target in atherosclerosis. Int. J. Biochem. Cell Biol. 40, 1095–1100. 10.1016/j.biocel.2008.01.004 18289916

[B19] Ho A. S. Chen C. H. Cheng C.-C. Wang C.-C. Lin H. C. Luo T. Y.. (2014). Neutrophil elastase as a diagnostic marker and therapeutic target in colorectal cancers. Oncotarget 5:473. 10.18632/oncotarget.1631 24457622 PMC3964222

[B20] Houghton A. M. Rzymkiewicz D. M. Ji H. B. Gregory A. D. Egea E. E. Metz H. E.. (2010). Neutrophil elastase-mediated degradation of IRS-1 accelerates lung tumor growth. Nat. Med. 16:219. 10.1038/nm.2084 20081861 PMC2821801

[B21] Karami E. Behdani M. Kazemi-Lomedasht F. (2020). Albumin nanoparticles as nanocarriers for drug delivery: Focusing on antibody and nanobody delivery and albumin-based drugs. J. Drug Deliv. Sci. Tec. 55:101471 10.1016/j.jddst.2019.101471

[B22] Kasperkiewicz P. Poreba M. Snipas S. J. Parker H. Winterbourn C. C. Salvesen G. S.. (2014). Design of ultrasensitive probes for human neutrophil elastase through hybrid combinatorial substrate library profiling. Proc. Natl. Acad. Sci. U.S.A. 111, 2518–2523. 10.1073/pnas.1318548111 24550277 PMC3932852

[B23] Kobayashi H. Ogawa M. Alford R. Choyke P. L. Urano Y. (2010). New strategies for fluorescent probe design in medical diagnostic imaging. Chem. Rev. 110, 2620–2640. 10.1021/cr900263j 20000749 PMC3241938

[B24] Korkmaz B. Horwitz M. S. Jenne D. E. Gauthier F. (2010). Neutrophil elastase, proteinase 3, and cathepsin G as therapeutic targets in human diseases. Pharmacol. Rev. 62, 726–759. 10.1124/pr.110.002733 21079042 PMC2993259

[B25] Liu H. W. Chen L. Xu C. Li Z. Zhang H. Zhang X. B.. (2018). Recent progresses in small-molecule enzymatic fluorescent probes for cancer imaging. Chem. Soc. Rev. 47, 7140–7180. 10.1039/C7CS00862G 30140837

[B26] Liu S. Y. Xiong H. Li R. R. Yang W. C. Yang G. F. (2019). Activity-based near-infrared fluorogenic probe for enabling in vitro and in vivo profiling of neutrophil elastase. Anal. Chem. 91, 3877–3884. 10.1021/acs.analchem.8b04455 30626182

[B27] Miranda-Apodaca J. Hananya N. Velázquez-Campoy A. Shabat D. Arellano J. B. (2019). Emissive enhancement of the singlet oxygen chemiluminescence probe after binding to bovine serum albumin. Molecules 24:2422. 10.3390/molecules24132422 31266247 PMC6651777

[B28] Mitra S. Modi K. D. Foster T. H. (2013). Special section on fluorescence molecular imaging honoring prof. roger tsien, a pioneer in biomedical optics: enzyme-activatable imaging probe reveals enhanced neutrophil elastase activity in tumors following photodynamic therapy. J. Biomed. Opt. 18:101314. 10.1117/1.JBO.18.10.101314 23897439 PMC3726228

[B29] Nelson D. L. Cox M. M. (2005). Lehninger Principles of Biochemistry, 4th Edn New York, NY: W. H. Freeman and Company 202–207.

[B30] Odyniec M. L. Sedgwick A. C. Swan A. H. Weber M. Tang T. M. S. Gardiner J. E.. (2018). ‘AND'-based fluorescence scaffold for the detection of ROS/RNS and a second analyte. Chem. Commun. 54, 8466–8469. 10.1039/C8CC04316G 29999509

[B31] Sadat Ebrahimi M. M. Voss Y. Schönherr H. (2015). Rapid detection of Escherichia coli via enzymatically triggered reactions in self-reporting chitosan hydrogels. ACS Appl. Mater. Interfaces 7, 20190–20199. 10.1021/acsami.5b05746 26322857

[B32] Schulz-Fincke A. C. Tikhomirov A. S. Braune A. Girbl T. Gilberg E. Bajorath J.. (2018). Design of an activity-based probe for human neutrophil elastase: implementation of the lossen rearrangement to induce förster resonance energy transfers. Biochemistry 57, 742–752. 10.1021/acs.biochem.7b00906 29286643

[B33] Sedgwick A. C. Dou W. T. Jiao J. B. Wu L. Williams G. T. Jenkins A. T. A.. (2018a). An ESIPT probe for the ratiometric imaging of peroxynitrite facilitated by binding to Aβ-aggregates. J. Am. Chem. Soc. 140, 14267–14271. 10.1021/jacs.8b08457 30277762

[B34] Sedgwick A. C. Gardiner J. E. Kim G. Yevglevskis M. Lloyd M. D. Jenkins A. T. A.. (2018b). Long-wavelength TCF-based fluorescence probes for the detection and intracellular imaging of biological thiols. Chem. Commun. 54, 4786–4789. 10.1039/C8CC01661E 29683468 PMC5944426

[B35] Sedgwick A. C. Han H. H. Gardiner J. E. Bull S. D. He X. P. James T. D. (2017). Long-wavelength fluorescent boronate probes for the detection and intracellular imaging of peroxynitrite. Chem. Commun. 53, 12822–12825. 10.1039/C7CC07845E 29143045

[B36] Sedgwick A. C. Han H. H. Gardiner J. E. Bull S. D. He X. P. James T. D. (2018c). The development of a novel AND logic based fluorescence probe for the detection of peroxynitrite and GSH. Chem. Sci. 9, 3672–3676. 10.1039/C8SC00733K 29780497 PMC5935063

[B37] Sedgwick A. C. Wu L. Han H. H. Bull S. D. He X. P. James T. D.. (2018d). Excited-state intramolecular proton-transfer (ESIPT) based fluorescence sensors and imaging agents. Chem. Soc. Rev. 47, 8842–8880. 10.1039/C8CS00185E 30361725

[B38] Shapiro S. D. Goldstein N. M. Houghton A. M. Kobayashi D. K. Kelley D. Belaaouaj A. (2003). Neutrophil elastase contributes to cigarette smoke-induced emphysema in mice. Am. J. Pathol. 163, 2329–2335. 10.1016/S0002-9440(10)63589-4 14633606 PMC1892384

[B39] Sheng Z. Hu D. Zheng M. Zhao P. Liu H. Gao D.. (2014). Smart human serum albumin-indocyanine green nanoparticles generated by programmed assembly for dual-modal imaging-guided cancer synergistic phototherapy. ACS Nano 8, 12310–12322. 10.1021/nn5062386 25454579

[B40] Sly P. D. Brennan S. Gangell C. de Klerk N. Murray C. Mott L.. (2009). Australian Respiratory Early Surveillance Team for Cystic Fibrosis (AREST-CF) Lung disease at diagnosis in infants with cystic fibrosis detected by newborn screening. Am. J. Respir Crit. Care Med. 180, 146–152. 10.1164/rccm.200901-0069OC 19372250

[B41] Sun Q. Li J. Liu W. N. Dong Q. J. Yang W. C. Yang G. F. (2013). Non-peptide-based fluorogenic small-molecule probe for elastase. Anal. Chem. 85, 11304–11311. 10.1021/ac402097g 24219095

[B42] Wang Y. Zagorevski D. V. Stenken J. A. (2008). *In situ* and multisubstrate detection of elastase enzymatic activity external to microdialysis sampling probes using LC– ESI-MS. Anal. Chem. 80, 2050–2057. 10.1021/ac702047w 18278883 PMC4717840

[B43] Wu D. Sedgwick A. C. Gunnlaugsson T. Akkaya E. U. Yoon J. James T. D. (2017). Fluorescent chemosensors: the past, present and future. Chem. Soc. Rev. 46, 7105–7123. 10.1039/C7CS00240H 29019488

[B44] Wu L. Han H. H. Liu L. Gardiner J. E. Sedgwick A. C. Huang C.. (2018). ESIPT-based fluorescence probe for the rapid detection of peroxynitrite ‘AND'biological thiols. Chem. Commun. 54, 11336–11339. 10.1039/C8CC06917D 30246201

[B45] Xiao T. Ackerman C. M. Carroll E. C. Jia S. Hoagland A. Chan J.. (2018). Copper regulates rest-activity cycles through the locus coeruleus-norepinephrine system. Nat. Chem. Biol. 14, 655–663. 10.1038/s41589-018-0062-z 29867144 PMC6008210

[B46] Yan H. Gao Q. Liu Y. Ren W. Shangguan J. Yang X. (2018). Poly (β-cyclodextrin) enhanced fluorescence coupled with specific reaction for amplified detection of GSH and trypsin activity. New J. Chem. 42, 17682–17689. 10.1039/C8NJ04325F

[B47] Yang C. Wang Q. Ding W. (2019). Recent progress in the imaging detection of enzyme activities *in vivo*. RSC Adv. 9, 25285–25302. 10.1039/C9RA04508B PMC9070033 35530057

