# Supplementary material for: Protein Encapsulation: A Nanocarrier Approach to the Fluorescence Imaging of an Enzyme-Based Biomarker
Source: Front Chem. 2020 Jun 3;8:389. doi: 10.3389/fchem.2020.00389 (PMC7283737; doi:10.3389/fchem.2020.00389)
Supplement: Supplementary file 1 [file Table_1.docx]

**Protein encapsulation: A nanocarrier approach to the fluorescence imaging of an enzyme-based biomarker**

Zhiyuan Jia^1‡^, Hai-Hao Han^2‡^, Adam C. Sedgwick^3‡^, George T. Williams^4^, Lauren Gwynne^4^, James T. Brewster II^3^, Steven D. Bull^4^, A. Toby A. Jenkins^4^, Xiao-Peng He*^2^, Holger Schönherr^1^*, Jonathan L. Sessler^3^* and Tony D. James^4^*

^1^Physical Chemistry I & Research Center of Micro and Nanochemistry and Engineering (Cμ), Department of Chemistry and Biology, University of Siegen, Adolf-Reichwein-Straße 2, 57076 Siegen, Germany.

^2^Key Laboratory for Advanced Materials and Joint International Research Laboratory of Precision Chemistry and Molecular Engineering, Feringa Nobel Prize Scientist Joint Research Center, School of Chemistry and Molecular Engineering, East China University of Science and Technology, 130 Meilong Rd., Shanghai 200237, China.

^3^Department of Chemistry, The University of Texas at Austin, 105 E 24th street A5300, Austin, TX 78712-1224, USA.

^4^Department of Chemistry, University of Bath, Bath, BA2 7AY, UK.

^‡^Equal contributions

**Email:**

t.d.james@bath.ac.uk, schoenherr@chemie.uni-siegen.de, xphe@ecust.edu.cn, Sessler@cm.utexas.edu

Table of Contents

1. General information S3
2. UV-Vis and fluorescence spectroscopy data S6
3. Cellular imaging and cell viability data S11
4. Experimental S13
5. NMR spectra S15
6. General information

**Materials.** Phosphate buffered saline (PBS, tablet), elastase from porcine pancreas (7 units/mg, E.C. 3.4.21.36), β-glucuronidase purified from *E. coli* (β-Gus, 694.3 units/mg, E.C.3.2.1.31), β-galactosidase from *E. coli* (β-Gal, 174 units/mg, E.C. 3.2.1.23), trypsin from porcine pancreas (1000-2000 BAEE unit/mg, E.C. 3.4.21.4), esterase from *Bacillus subtilis* (29 units/mg, E.C. [3.1.1.1](about:blank)), α-chymotrypsin from bovine pancreas (≥40 units/mg, E.C. 3.4.21.1), α-glucosidase from *Saccharomyces cerevisi* (≥16.13 units/mg, E.C. 3.2.1.20), glutathione and rhodamine chloride 110 were purchased from Sigma-Aldrich. Hydrogen peroxide (H_2_O_2_, 30%, Roth), bovine serum albumin (BSA, Acros Organics), DMSO (99% pure, Merck), were purchased from the listed suppliers. Milli-Q water, obtained from a Millipore Direct Q 8 system (Millipore, Schwalbach, Germany) with a resistivity of 18 MΩ cm, was used for preparation of all aqueous media. Syringe filters consisted of 0.45 µm PTFE membranes (VWR, USA).

# UV-Vis spectroscopy

Spectra were recorded on a Varian Cary 50 Bio spectrometer (Mulgrave, Victoria, Australia) over the 250 – 600 nm wavelength range at a scan rate of 300 nm/minute. A quartz cell with 1 cm path length (SUPRASIL, Hellma Analytics, Germany) was used for transmission measurements. All spectra were zeroed at 600 nm.

# Fluorescence spectroscopy

Measurements were carried out either on a Varian Cary Eclipse spectrometer (Mulgrave, Victoria, Australia) or a microplate reader (Tecan SAFIRE, Tecan, Switzerland) at 25°C. Fluorescence spectra obtained with the spectrometer were measured at a scan rate of 120 nm/minute and a resolution of 2.5 nm for the excitation and emission, using a 1 cm path-length quartz cell (SUPRASIL, Hellma Analytics, Germany). Fluorescence spectra, which were recorded on the microplate reader, were performed using 96 well plates (black, polystyrene, flat bottom, Greiner Bio-One, Austria) and a sample holder with a clear Viewseal® sealer (Greiner Bio-One, Austria). The bandwidth was 12 nm for the both excitation and emission studies. The gain parameter was manually set to 70.

**Enzymatic reactions of ACS-HNE in solution**. 10 µL of **ACS-HNE** (50 µM in DMSO) substrate solution were deposited in a black 96-well microplate, followed by the addition of 90 µL of buffered elastase solution (phosphate buffered saline, PBS, pH 7.40) at various enzyme concentrations. The microplate was then covered with a clear Viewseal® sealer. The kinetics of the enzymatic reactions were monitored by recording the emission intensity at a wavelength of 527 nm (excitation wavelength: 496 nm) using a microplate reader. Monitoring was carried out over the course of 24 h with time intervals of 1 min. The baseline was recorded using a mixed solution consisting of 10 μL of **ACS-HNE** (50 μM in DMSO) and 90 μL of PBS (pH 7.40). All fluorescence intensity values were corrected to zero at t = 0.

**Selectivity tests**. 90 µL of each buffered enzyme solution subject to testing, hydrogen peroxide (H_2_O_2_), as well as glutathione (GSH) solutions at defined concentrations (in PBS, pH 7.40) were added to 10 µL of the **ACS-HNE** (50 µM, in DMSO) substrate solution in a black 96-well microplate. The microplate was subsequently covered with a clear Viewseal® sealer.

**Cell culture studies**. RAW 264.7 cells were maintained in a Dulbecco’s Modified Eagle’s Medium (Invitrogen, Carlsbad, CA, USA) supplemented with 10 % fetal bovine serum (Gibco, Gland Island, NY, USA) in a humidified atmosphere consisting of 5 % CO_2_ and 95 % air at 37 °C. The cells were split when they reached 90 % confluency.

**Fluorescence cell imaging.** Cells were seeded on a black 96-well microplate with an optically clear bottom (Greiner bio-one, Germany) and allowed to incubate overnight. **ACS-HNE** was mixed with BSA at a molar ratio of 1:5 (**ACS-HNE**/**BSA** = 20 μM/100 μM) prior to cell imaging. The **ACS-HNE**/**BSA** hybrid was added to the cells and incubated for 30 min. Then, human neutrophil elastase (154.4 nM, 100 μL) was added to the cell mixture and incubated for 3 h. Then, the cells were washed with PBS three times. Fluorescence images were recorded using an Operetta high-content imaging system (Perkin Elmer, US) with an excitation channel of 460-490 nm and an emission channel of 500-550 nm. The fluorescence intensity was quantified and plotted using the Columbus analysis system (Perkin Elmer, US).

**Cell viability assay.** Cells were plated overnight on a 96-well plate in growth medium. After seeding, cells were treated with **ACS-HNE** at different concentrations for 24 h. Then, a MTS/PMS (20:1, Promega Corp, 10 μL) solution was added to each well containing 100 μL of the growth medium. After incubation at 37 °C under 5% CO_2_ for 2 h, the absorbance of the solutions was measured at 490 nm with an M5 microplate reader (Molecular Device, USA). The optical density of the result in the MTS assay was considered as being directly proportional to the number of viable cells.

1. UV-Vis and fluorescence spectroscopy data


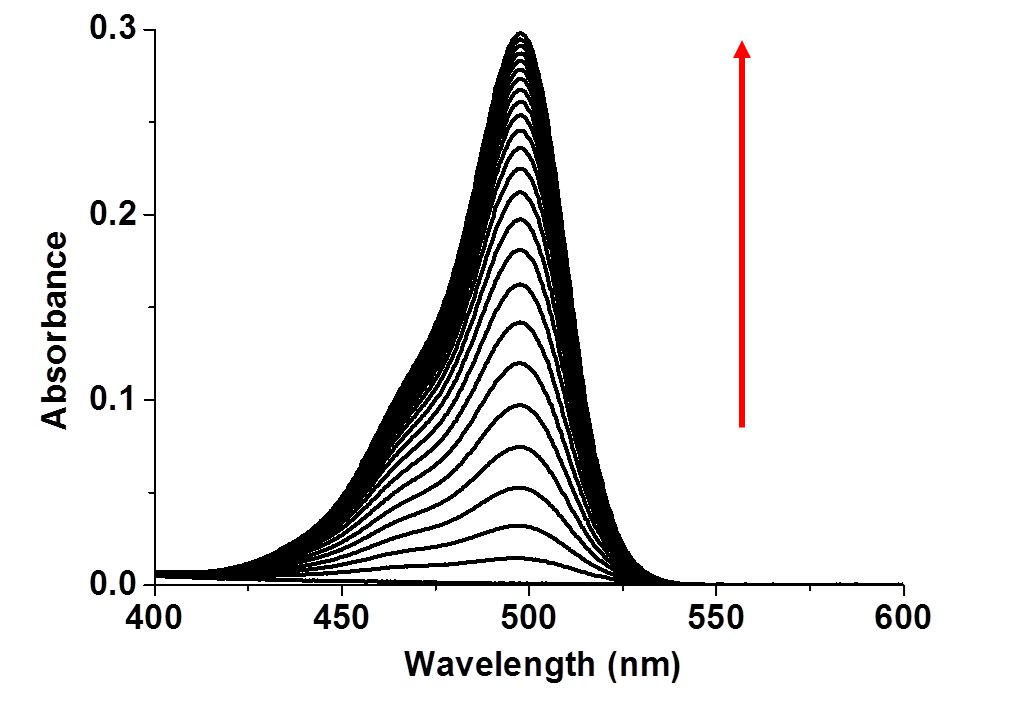


**Figure S1 –** UV-Vis spectra (Varian Cary 50 Bio spectrometer) of **ACS-HNE** (5 µM) over time (24 h) in buffered elastase enzyme (2 μM) solution (in PBS buffer, pH = 7.40). Measurements taken in intervals of 1 h.

**
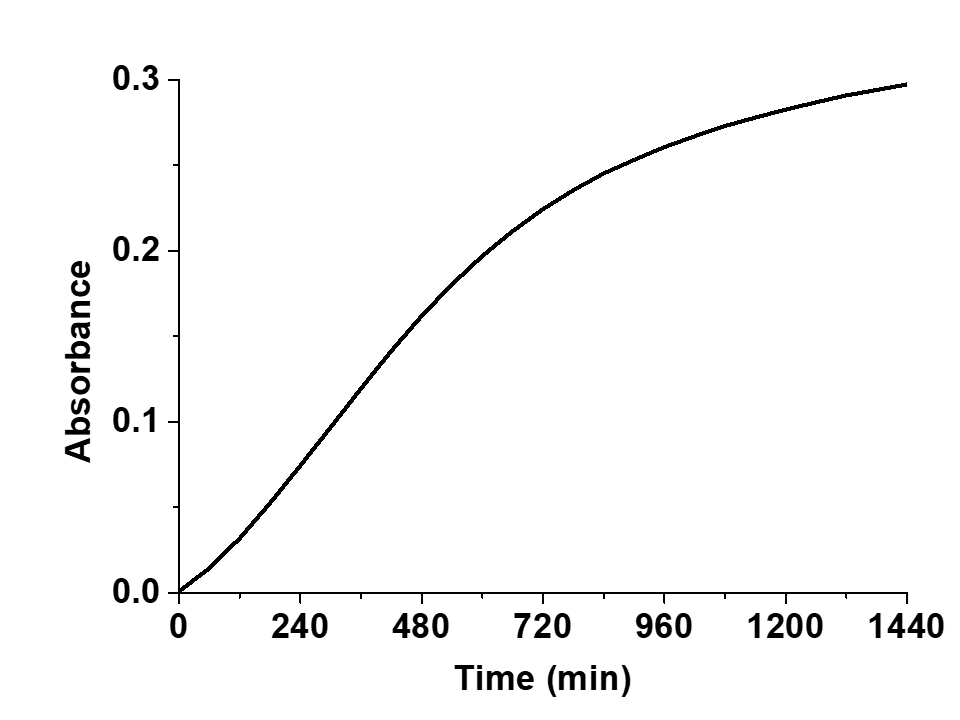
**

**Figure S2 –** Changes in the absorbance intensity at 498 nm (Varian Cary 50 Bio spectrometer) plotted against time for the reaction of **ACS-HNE** (5 µM) with buffered elastase enzyme solution (2 μM in PBS buffer, pH = 7.40).


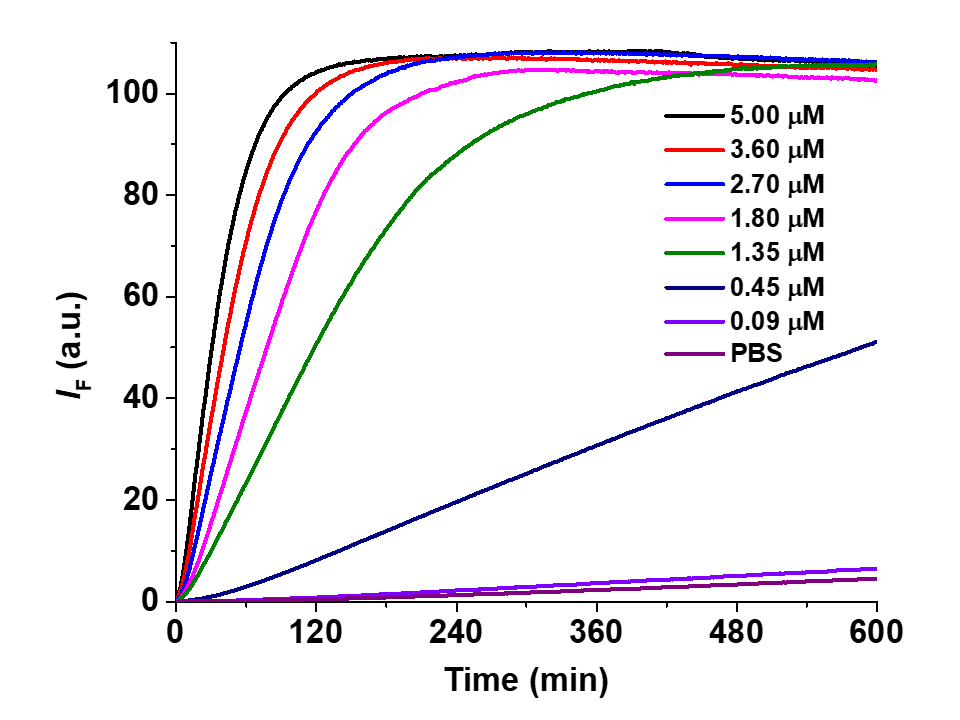


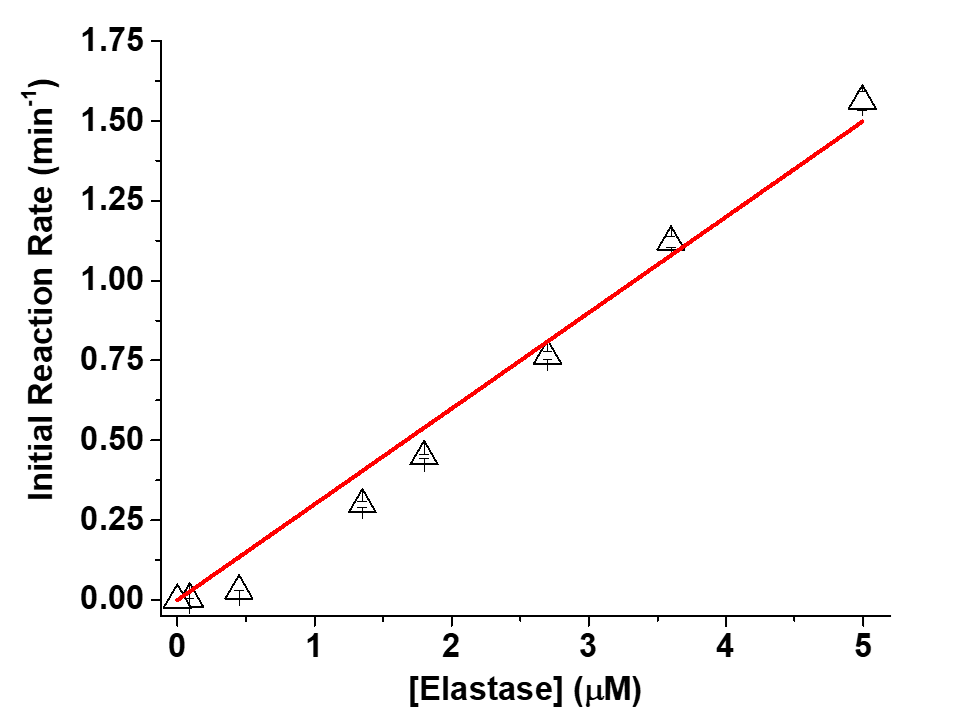
**Figure S3 –** Changes in fluorescence intensity (Tecan microplate reader) at 527 nm plotted against time for the reaction of **ACS-HNE** (5 µM) with buffered elastase enzyme (listed concentrations) solution (in PBS buffer, pH = 7.40); λ_ex_ = 496 nm.

**Figure S4** – Plots of the calculated initial apparent reaction rate during the first 30 min of the enzymatic reaction versus the enzyme concentration. The initial apparent rate is defined as the slope, as obtained from a linear least squares fit, at a given concentration of buffered enzyme solution. The red line is the resulting fit: Initial reaction rate = 0.30 (± 0.01) min^-1^ µM^-1^ × [Elastase]. This experiment was repeated in triplicate, and the error bars represent standard deviation.


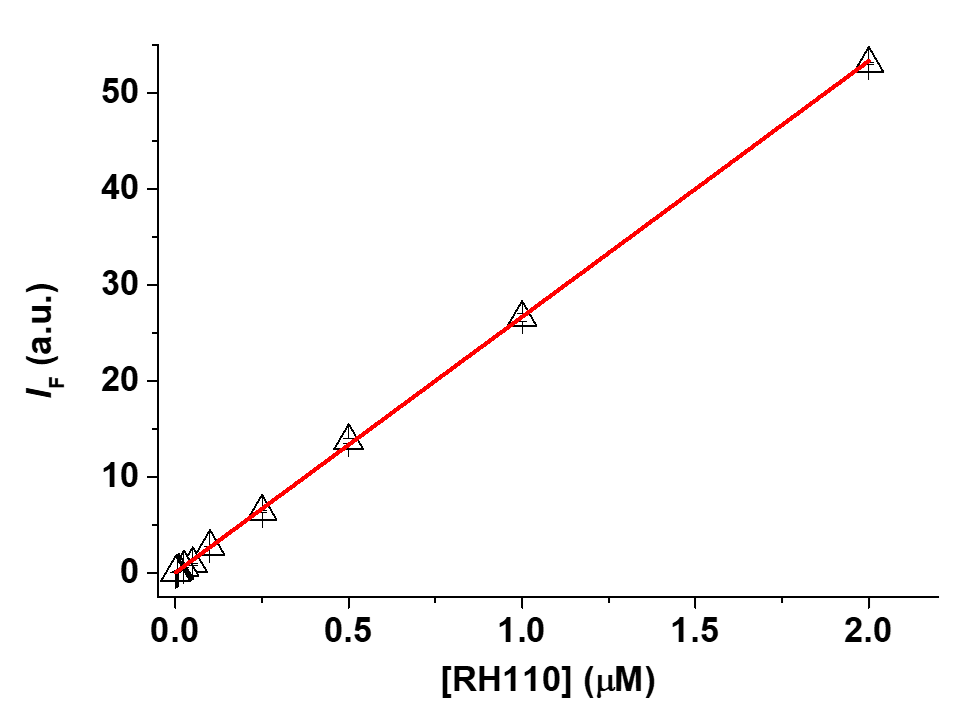

**Figure S5 –** Fluorescence intensity (Tecan microplate reader) at 527 nm for rhodamine 110 (RH 110) versus its concentration in PBS buffer (pH = 7.40; λ_ex_ = 496 nm, 100 µL). The red line corresponds to a linear least squares fit of the data. The calculated LoD for RH110 is defined as the mean of the background (intercept of the fit) plus three times the standard deviation of the background. The LoD of RH110 was calculated as 1.7 nM. The red line corresponds to I_F_ = 26.655 (± 0.165) µM^-1^ × [RH110] + 0.050 (± 0.015). This experiment was repeated in triplicate, and the error bars represent standard deviation.


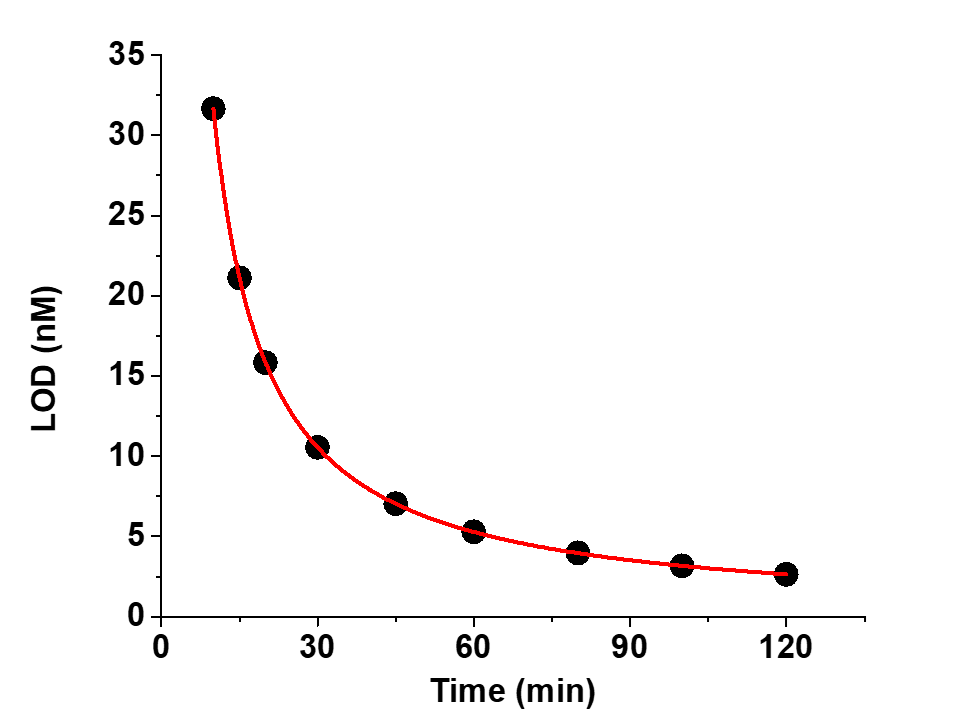


**Figure S6 –** Plots of the LOD for elastase versus the reaction time in the presence of **ACS-HNE** solution. The data were fitted with: LOD_(Elastase)_ = 317 (± 9) nM^.^min^-1^× t^-1^.

**Figure S7** - Fluorescence intensity changes of **ACS-HNE** (5 µM) against time determined in the presence of various biological analytes. Elastase (black line) – 2.000 µM (0.36 U/mL) provided a clear increase in fluorescence intensity, while α-glucosidase – 0.400 µM (0.4 U/mL), α-chymotrypsin – 0.400 µM (0.4 U/mL), β-glucuronidase –8 nM (4 kU/mL), trypsin –0.011 µM (0.4 U/mL), esterase – 0.082 µM (0.4 U/mL), β-galactosidase – 0.006 µM (0.4 U/mL), bovine serum albumin (BSA) – 0.800 µM, hydrogen peroxide (H_2_O_2_) – 0.1 mM, and glutathione (GSH) – 1 mM in PBS buffer (pH = 7.40); *λ*_ex_ =496; *λ*_em_ = 527 nm produced minimal response.

The reader should take note that for the selectivity studies, **ACS-HNE** was compared with each enzyme’s activity (U/mL) rather than molar concentrations.

1. Cellular imaging and cell viability data


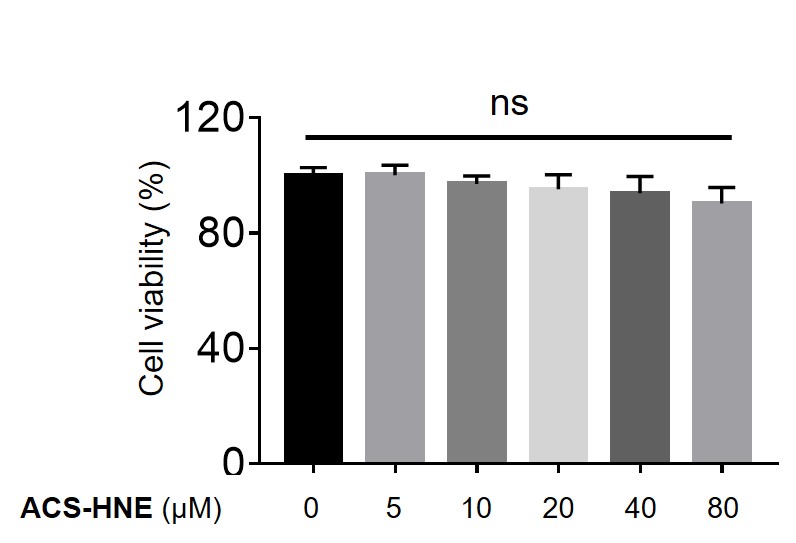


**Figure S8 –** Cell viability assay of **ACS-HNE**. Measurements were taken after 24 hours. (ns indicates no significant difference compared to control of 0 µM of **ACS-HNE**). The experiment was conducted in triplicate, and error bars indicate standard deviation.


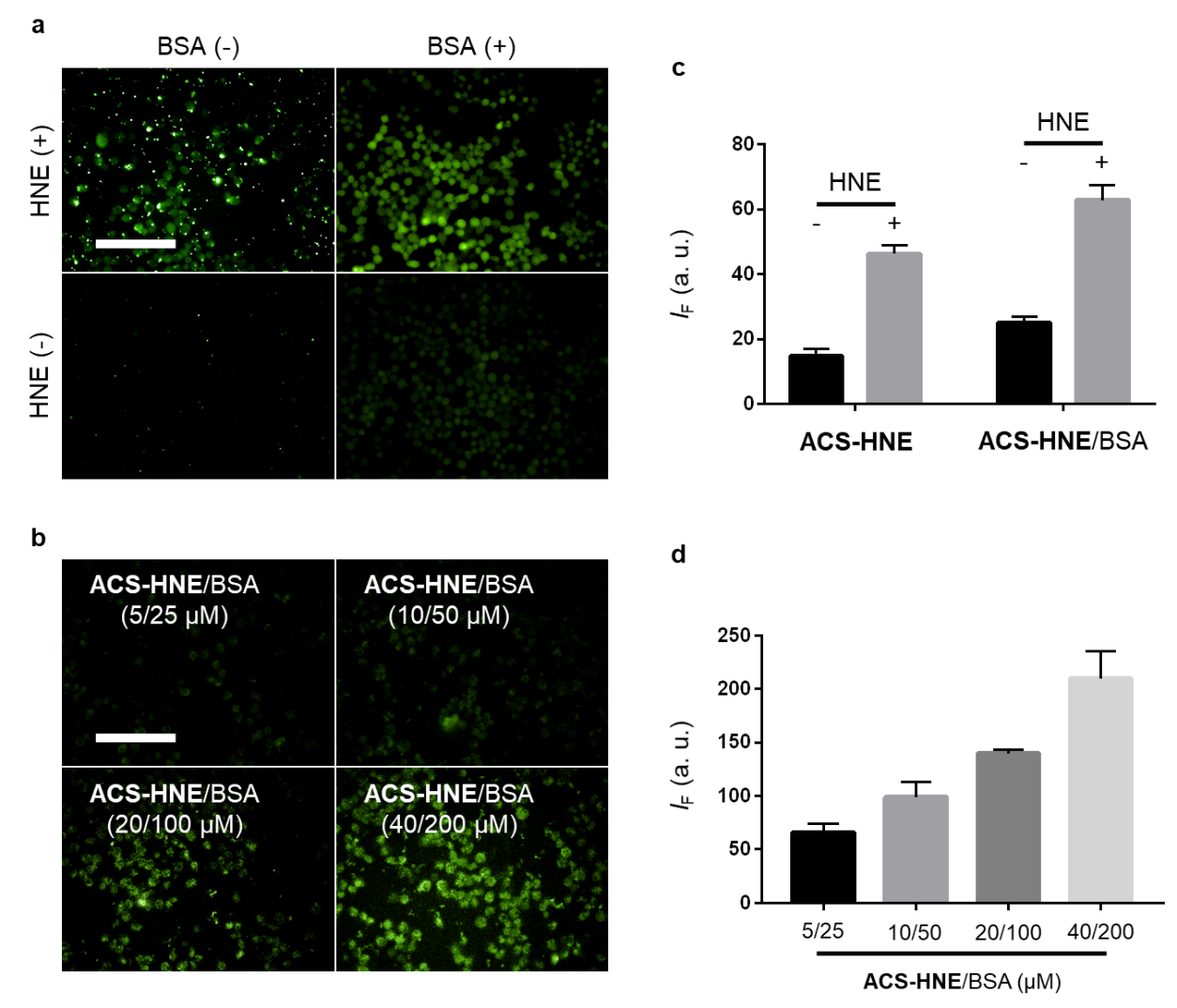


**Figure S9 –** Widefield fluorescence images (a) and intensity histograms (c) of Raw 264.7 cells incubated with **ACS-HNE** (20 μM) in the absence or presence of bovine serum albumin (BSA) (100 μM) with or without a subsequent addition of human neutrophil elastase (HNE, 154.4 nM, 100 μL). Widefield fluorescence images (b) and intensity histograms (d) of Raw 264.7 cells incubated with increasing concentrations of **ACS-HNE** and BSA and a subsequent addition of HNE (154.4 nM, 100 μL). Excitation and emission wavelengths for **ACS-HNE** are 460-490 nm and 500-550 nm, respectively. Error bars represent standard deviation. (n = 3).

1. Experimental

**Scheme S1 –** Synthetic procedure used to prepare **ACS-HNE**

***N*,*N'*-(3-oxo-3H-spiro[isobenzofuran-1,9'-xanthene]-3',6'-diyl)bis(2,2,3,3,3-pentafluoropropanamide)**

Rhodamine 110 (0.10 g, 0.30 mmol) in DMF (5 mL) was cooled to 0 ^o^C followed by the portion-wise addition of NaH-60 % in mineral oil (0.036 g, 0.91 mmol). The reaction mixture was left to stir for 15 min and pentafluoropropionic anhydride (0.3 mL, 1.51 mmol) was subsequently added dropwise. The reaction mixture was stirred for 4 h before being quenched with H_2_O (25 mL). The aqueous solution was then extracted EtOAc (2 x 50 mL) and the combined organics were washed with H_2_O (25 mL) and brine (25 mL) before being dried (MgSO_4_). The solvent was removed *in vacuo* to afford the crude product, which was purified via column chromatography (20:80 to 50:50 (EtOAc:petroleum ether)) to afford the title compound as a white solid (0.080 g, 0.13 mmol, 43 %). M.P. = 183 – 186 ^o^C; ^1^H NMR (500 MHz, DMSO-*d*_6_) δ 11.59 (s., 2 H, N-*H*), 8.06 (dd, *J* = 2.2, 7.6 Hz, 1 H, Ar*H*), 7.86 (m, 2 H, Ar*H*), 7.81 (td, *J* = 2.4, 7.3 Hz, 1 H, Ar*H*), 7.75 (td, *J* = 2.7, 7.5 Hz, 1 H, Ar*H*), 7.46 - 7.42 (m, 2 H, Ar*H*), 7.32 (dd, *J* = 2.7, 7.6 Hz, 1 H, Ar*H*), 6.92 (dd, *J* = 3.4, 8.8 Hz, 2 H, Ar*H*); ^13^C NMR (126 MHz, DMSO-d_6_) δ 169.0, 156.1 (t, *J* = 25.75 Hz), 152.9, 150.9, 138.9, 136.4, 130.9, 129.3, 125.8, 125.4, 124.4, 118.21 (qt, *J* = 287.06 Hz, *J* = 35.29 Hz), 117.8, 116.2, 109.3, 106.86 (tq, *J* = 267.04 Hz, *J* = 38.15 Hz), 81.3; ^19^F NMR (470 MHz, DMSO-*d*_6_) δ -82.09; -121.16; I.R (thinfilm) ν max (cm^-1^): 1712.71, 1749,76 (C=O); HRMS (FTMS-NSI): m/z calculated for C_26_H_12_F_10_N_2_O_5_ requires 623.0659 for [M+H]^+^, found 623.0653

1. NMR spectra

***N*,*N'*-(3-oxo-3H-spiro[isobenzofuran-1,9'-xanthene]-3',6'-diyl)bis(2,2,3,3,3-pentafluoropropanamide) -** ^1^H NMR spectrum (500 MHz, DMSO-*d*_6_)

***N*,*N'*-(3-oxo-3H-spiro[isobenzofuran-1,9'-xanthene]-3',6'-diyl)bis(2,2,3,3,3-pentafluoropropanamide) –** ^13^C NMR spectrum (125.7 MHz, DMSO-*d*_6_)

***N*,*N'*-(3-oxo-3H-spiro[isobenzofuran-1,9'-xanthene]-3',6'-diyl)bis(2,2,3,3,3-pentafluoropropanamide) –** ^19^F NMR spectrum (470 MHz, DMSO-*d*_6_)
